# Social learning exploits the available auditory or visual cues

**DOI:** 10.1038/s41598-020-71005-x

**Published:** 2020-08-24

**Authors:** Nihaad Paraouty, Joey A. Charbonneau, Dan H. Sanes

**Affiliations:** 1 Center for Neural Science, New York University, 4 Washington Place, New York, NY 10003 USA; 2 Department of Psychology, New York University, New York, NY 10003 USA; 3 Department of Biology, New York University, New York, NY 10003 USA; 4 Neuroscience Institute, NYU Langone Medical Center, New York University, New York, NY 10003 USA

**Keywords:** Social evolution, Social behaviour

## Abstract

The ability to acquire a behavior can be facilitated by exposure to a conspecific demonstrator. Such social learning occurs under a range of conditions in nature. Here, we tested the idea that social learning can benefit from any available sensory cue, thereby permitting learning under different natural conditions. The ability of naïve gerbils to learn a sound discrimination task following 5 days of exposure adjacent to a demonstrator gerbil was tested in the presence or absence of visual cues. Naïve gerbils acquired the task significantly faster in either condition, as compared to controls. We also found that exposure to a demonstrator was more potent in facilitating learning, as compared to exposure to the sounds used to perform the discrimination task. Therefore, social learning was found to be flexible and equally efficient in the auditory or visual domains.

## Introduction

The acquisition of new skills can be facilitated by social experience, usually by exposure to a conspecific performing a well-defined behavior^1–5^. Such social learning is found in many species, and accelerates the acquisition of many behaviors, from eating habits and predator avoidance, to vocal learning and aural communication^6–17^. However, social learning may occur under natural conditions in which some sensory cues are absent (e.g., in the dark). This raises the question of whether animals can benefit from switching the sensory cues that they use to learn from their conspecifics.

Social learning is particularly important in the early stages of life, especially during interactions with siblings or parents^18–20^. Hence, social learning has often been studied in animals that live in social groups (e.g., monkeys^18^; whales^21^) or display regular social interactions (e.g., rats^22^). Mongolian gerbils (*Meriones unguiculatus*) live as social families in which a founder pair breeds monogamously^23^, making them well-suited for the study of social learning. Furthermore, animals rely heavily on auditory cues when visibility is diminished^24,25^. This is true for many species, including the Mongolian gerbil, that live in underground burrows and are most active at night^26–28^. Here, we test the hypothesis that gerbils exploit the available sensory cues to learn a behavioral task, thereby permitting social learning to occur under challenging environmental conditions.

While learning through visual exposure (observation) has been well studied^22,29–31^, the acquisition of complex behaviors typically depends on multiple sensory cues from the environment and from social interactions. For instance, juvenile songbirds can learn elements of a species-specific vocalization through auditory exposure alone, but learning is enhanced by the presence of an adult tutor^32–37^. Similarly, filial imprinting involves many sensory and social cues^38^, but exposure to an auditory cue alone is sufficient to maintain or induce the behavior^39,40^. However, it is uncertain whether visual and auditory information are interchangeable when animals learn an identical task in a social context. For instance, a task requiring spatial information could be acquired with high resolution through either listening to, or watching, a conspecific demonstrator. Here, we assessed whether naïve gerbils could learn equally from social experience when either visual or auditory cues were unavailable.

In the current study, social learning refers to delayed imitation, whereby a naïve animal capitalizes on the presence and behavior of a conspecific to adapt its own behavior^5^. First, we showed that naïve observer gerbils can acquire a sound discrimination task significantly faster following 5 days of exposure with a performing demonstrator gerbil, as compared to separate controls for social and/or test cage exposure. To test the necessity of visual information during social learning, we removed visible cues with an opaque divider during the social exposure. These animals learned the task at a similar rate, suggesting that in the absence of visual information, gerbils can still capitalize on the remaining information to learn in a social context.

## Results

### Task acquisition is facilitated by observing a trained demonstrator

We first asked whether naïve gerbils could learn an auditory Go-Nogo task by observing a trained *demonstrator* gerbil perform. All demonstrator gerbils were trained by the experimenters to perform a Go-Nogo amplitude modulation (AM) discrimination task (n = 37; see “Methods”). Experimenter training of demonstrators required on average 15.9 days ± 0.17 days (mean ± SEM; see Supplementary Fig. 1). Once trained, demonstrator gerbils initiated each trial by entering a nose-poke, and discriminated between a 12 Hz AM stimulus (Go) that signaled the availability of a food reward and a 4 Hz AM stimulus (Nogo) that signaled the absence of a reward. Following Go trials, responses were scored as a ‘Hit’ when animals correctly approached the food tray for a reward (see Supplementary Fig. 1b). Following Nogo trials, responses were scored as a ‘False Alarm’ when animals incorrectly approached the food tray to seek a reward (see “Methods” for details). To qualify as a demonstrator, gerbils had to reach a performance metric, dprime (d’ = z(Hit rate) – z(False Alarm rate)) > 1.5 for 3 consecutive days.

**Figure 1:**
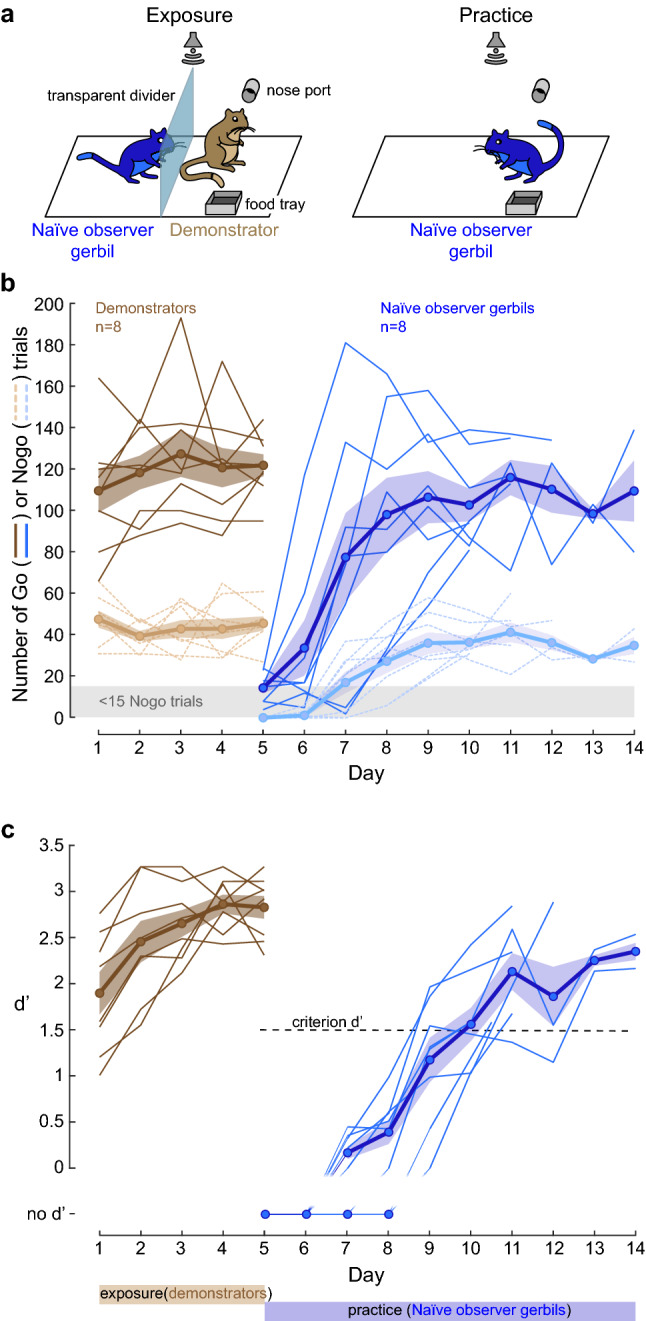
Social learning of a Go-Nogo task. (**a**) A naïve observer gerbil (blue) was separated from a performing demonstrator gerbil (cagemate, brown) by a transparent divider. The demonstrator was previously trained by the experimenters (see “Methods” for details). Right panel: Following five days of exposure, the practice phase began during which the naïve observer gerbil was permitted to practice the task on its own. (**b**) Individual (thin lines) and mean (thick lines) ± standard error of the mean (SEM) of overall number of Go (dark color) and Nogo trials (pale color) performed by the demonstrators during the 5 days of exposure (brown), and by the naïve observer gerbils during the practice sessions (blue). (**c**) Individual (thin lines) and mean ± SEM (thick lines) performance d’ values of the demonstrators during the 5 days of exposure (brown) and of the naïve observer gerbils during the practice sessions (blue).

Each experiment began by placing a naïve observer gerbil adjacent to a performing demonstrator gerbil for 5 consecutive days (Fig. 1a, left: exposure phase and Supplementary video 1). The demonstrator and naïve observer gerbil compartments were separated by a transparent divider, and the naïve observer gerbil had access to all sensory cues, whether they emanated from the demonstrator or the experimental apparatus (i.e., the Go and Nogo sounds). Immediately after the fifth day of exposure, each naïve observer gerbil was permitted to practice the task on its own (Fig. 1a, right). Figure 1b,c illustrate the performance of both the demonstrators (n = 8, brown, days 1 to 5) and the naïve observer gerbils (n = 8, blue, days 5 to 14). All demonstrator gerbils were performing > 60 Go trials and > 20 Nogo trials in each exposure session (Fig. 1b, brown). For the practice sessions of the naïve observer gerbil, Nogo trials were introduced when the naïve observer initiated and responded correctly to > 25 Go trials. We computed the sensitivity metric, d’, for all sessions during which a naïve observer gerbil performed > 15 Nogo trials. On average, the naïve observer gerbils required 6.0 ± 0.93 days to perform the task at a criterion d’ of 1.5 (Fig. 1c, blue square).

**Figure 2:**
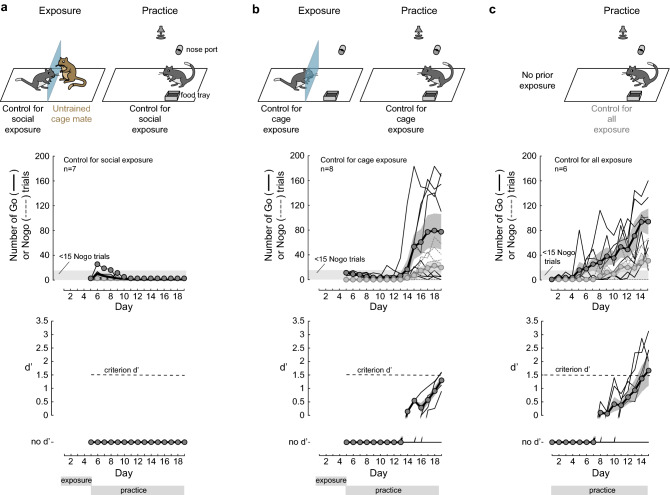
Delayed or no task acquisition in the absence of exposure to a performing demonstrator. (**a**) Control for social exposure: Naïve animals were exposed to an untrained and non-performing cage mate for 5 days. Following five days of exposure, the practice phase began, with 15 daily sessions (top panel). The individual (thin lines) and mean ± SEM (thick lines) overall number of Go (dark color) and Nogo trials (pale color) performed by the control gerbils (middle panel). The individual (thin lines) and mean ± SEM (thick lines) performance d’ values of the control gerbils during the 15 practice sessions (bottom panel). None of the controls for social exposure gerbils performed a sufficient number of Nogo trials (> 15) for d’ computation. (**b**) Control for cage exposure: Naïve animals were exposed to the test cage for 5 days prior to 15 practice sessions. Four of these 8 controls did not perform > 15 Nogo trials and no d’ could be computed. Only two of these controls reached a criterion d’ of 1.5 by day 15. (**c**) Control for all exposure: Naïve animals were provided with 15 practice sessions without any prior exposure to the test cage or to a cage mate. Two of these controls did not perform > 15 Nogo trials and no d’ could be computed. The remaining four animals all reached the criterion d’ within the 15 practice sessions.

Three control experiments were performed to determine whether social exposure alone and/or cage exposure could influence the rate of task acquisition. It is important to note that the control groups presented here, are each quite different from the ‘demonstrators’ that were individually hand-trained by the experimenters to perform the task (Supplementary Fig. 1; see Methods for more details on the experimenter-training procedure). First, to determine whether prior social exposure with a cage mate was sufficient to influence learning, naïve control gerbils (n = 7) were placed adjacent to a cage mate for 5 consecutive days, separated by a transparent divider (Fig. 2a, top left). The cage mate was untrained and did not perform a task during these five days. Following the five exposure sessions, each control gerbil for social exposure was allowed to practice the task for 15 daily sessions (Fig. 2a, top right). None of these controls reached a criterion d’ of 1.5 within the 15 practice days. In fact, each of them performed < 20 Go trials during each of the 15 practice sessions, which was significantly less than the naïve observer gerbils (comparison of total number of trials, Steel–Dwass non-parametric comparison, *p* = 0.001; Bayes Factor: BF_10_ = 267.7). These results are consistent with the interpretation that gerbils learn from observing conspecifics. In this case, they learned to remain passive.

Second, to determine whether prior exposure to the test cage was sufficient to influence learning, another group of naïve control gerbils (n = 8) was placed adjacent to an unoccupied demonstrator compartment, separated by a transparent divider, for 5 consecutive days (Fig. 2b, top left). Following the five cage exposure sessions, each control gerbil for test cage exposure was allowed to practice the task for 15 daily sessions (Fig. 2b, top right). Four of these 8 controls performed < 20 Go trials during each of the 15 sessions. The remaining controls performed significantly less trials than the naïve observer gerbils (comparison of total number of trials, Steel–Dwass non-parametric comparison, *p* = 0.008; BF_10_ = 10.1) and only two of these control gerbils reached a criterion d’ of 1.5 by day 15 (Fig. 2a, right panel).

Third, to control for prior exposure to both a cage mate and the test cage, a third group of naïve control gerbils (n = 6) received no exposure before practicing the task (Fig. 2c, top left). Each control gerbil with no prior exposure was allowed to practice the task for 15 daily sessions (Fig. 2c, top right). Two of the 6 controls performed < 20 Go trials during each of the 15 sessions. The remaining four controls performed a similar number of trials as the naïve observer gerbils (comparison of total number of trials, Steel–Dwass non-parametric comparison, *p* = 0.551; BF_10_ = 0.8) but required significantly more days to reach a criterion d’ of 1.5 (14.0 days ± 1.15 days, Steel–Dwass non-parametric comparison, *p* = 0.007; BF_10_ = 7.6e + 5).

Since the control gerbils for social (Fig. 2a) and time exposure (Fig. 2b) initiated less overall trials in comparison to the naïve observer gerbil group, we asked whether their exploratory behavior was suppressed. By analyzing video footage obtained during practice sessions, we found that the controls for social exposure displayed a significant reduction of total distance travelled in comparison to all other groups (Supplementary Fig. 2b; Kruskal–Wallis H test, X^2^(3) = 10.82, *p* = 0.012; Holm-Bonferroni-corrected post hoc comparisons, *p* < 0.05). In addition, the three control groups showed a significant reduction of exploration of the food tray and the nose port, as compared to the naïve observer gerbils (comparison of normalized number of occurrences at food tray and nose port; Supplementary Fig. 2c; Kruskal–Wallis H test, X^2^(3) = 8.74, *p* = 0.033; post hoc comparisons, *p* < 0.05). Moreover, the controls for social exposure spent significantly less time at the food tray and the nose port, as compared to the other two control groups (post hoc comparisons, *p* < 0.05). These findings suggest that social exposure with a non-performing conspecific was detrimental to task acquisition.

Finally, we considered the possibility that social learning was facilitated by the fact that naïve observer gerbils (from Fig. 1) were cagemates with their demonstrators. Sharing a home-cage could potentially give rise to exchange of odors and food^41^. In addition, gerbils communicate through vocalizations^42^, which may in part enhance learning during the exposure sessions. Here, naïve observer gerbils were exposed to same-sex but non-cagemate demonstrators for five daily sessions. The naïve observer gerbils were permitted to practice the task immediately following the final exposure session. The results showed no significant difference in learning rate between naïve observer gerbils when exposed to either cagemate or non-cagemate demonstrators (see Supplementary Fig. 3b; Steel–Dwass non-parametric comparison, *p* = 1.00; BF_10_ = 0.45). Although this experiment does not address the fact that there might still be exchange of information in the home cage, as all naïve observer animals were undergoing the exposure and practice sessions, these results suggest that social learning seems to be occurring primarily during the exposure sessions rather than in the home cage.

### Social learning can occur in the absence of visual cues

**Figure 3:**
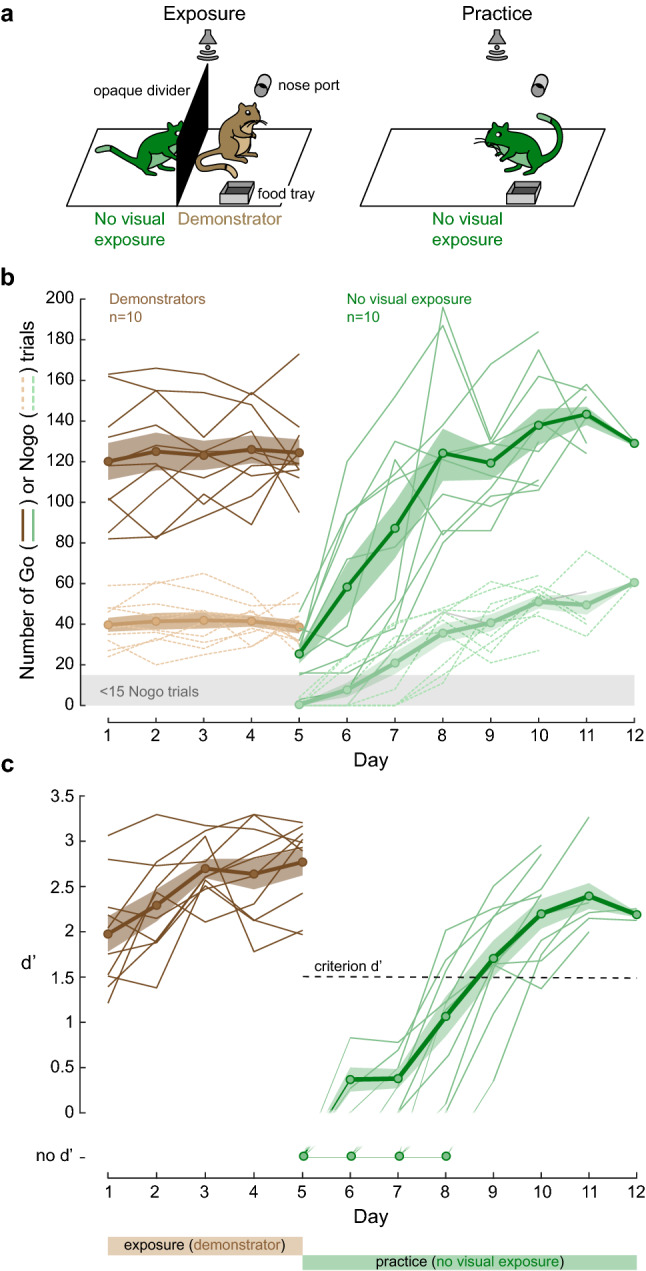
Visual cues are not crucial for social learning of an auditory Go-Nogo task. (**a**) A naïve gerbil (No visual exposure, green) was separated from a performing demonstrator gerbil (cagemate, brown) by an opaque divider. Right panel: Following five days of exposure, the practice phase began during which the No visual exposure gerbil was permitted to practice the task on its own. (**b**) Individual (thin lines) and mean ± SEM (thick lines) of overall number of Go (dark color) and Nogo trials (pale color) performed by the demonstrators during the 5 days of exposure (brown), and by the No visual exposure gerbils during the practice sessions (green). (**c**) Individual (thin lines) and mean ± SEM (thick lines) performance d’ values of the demonstrators during the 5 days of exposure (brown lines) and of the No visual exposure gerbils during the practice sessions (green lines).

The first experiment suggested that gerbils display social learning, but it did not distinguish between the sensory modalities that were required. To test whether social learning could occur in the absence of visual cues, we separated naïve gerbils from performing demonstrators with an opaque divider (Fig. 3a, left). These No visual exposure gerbils were exposed to a performing demonstrator during 5 daily sessions, and were permitted to practice the task immediately following the final exposure session (Fig. 3a, right). Figure 3b,c illustrate the performance of both the demonstrators (n = 10, brown, days 1 to 5) and the No visual exposure gerbils (n = 10, green, days 5 to 12). On average, the No visual exposure group required 4.9 ± 0.4 days to perform the task at a criterion d’ of 1.5. The performance of No visual exposure gerbils did not differ significantly from that displayed by the naïve observer gerbils (Steel–Dwass non-parametric comparison, *p* = 0.213; BF_10_ = 1.9; see also Fig. 5a,b). This indicates that visual cues were dispensable for such social learning.

### Socially-mediated learning leads to faster task acquisition as compared to statistical learning

**Figure 4:**
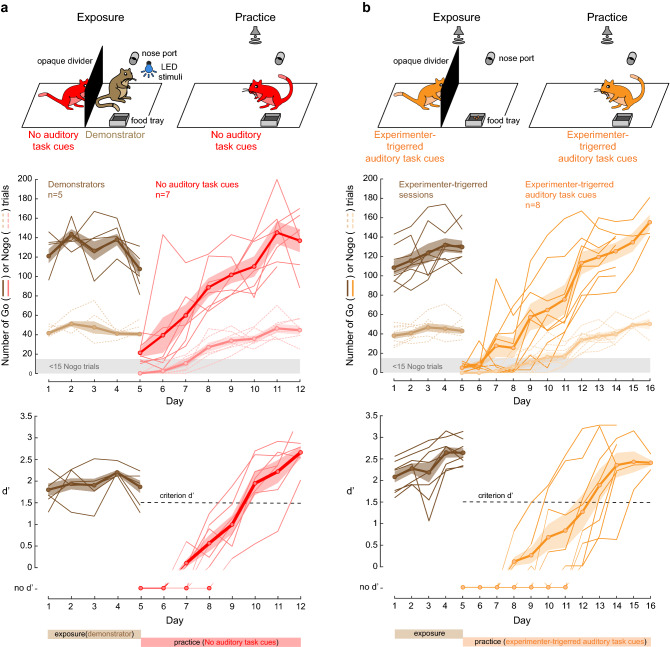
Social cues or exposure to auditory task sounds are sufficient for task acquisition. (**a**) Demonstrators were trained by the experimenters to perform a *visual* discrimination task (see “Methods” for details). Naïve gerbils (red) were separated from the trained and performing visual demonstrator gerbil (cagemate, in brown) by an opaque divider, blocking access to visual cues as well as the LED stimuli. Thus, no auditory task cues were present during the exposure sessions (i.e., Go and Nogo sound stimuli). Following five days of exposure, the practice phase began during which the No auditory task cues gerbil was permitted to practice the *auditory* discrimination task. (**b**) Naïve gerbils (orange) were separated from an unoccupied demonstrator’s compartment by an opaque divider and were exposed to experimenter-triggered auditory task cues (Go and Nogo stimuli), as well as pellet delivery and time-outs. Following five days of exposure to only the auditory task cues and the task contingencies, the practice phase began.

**Figure 5:**
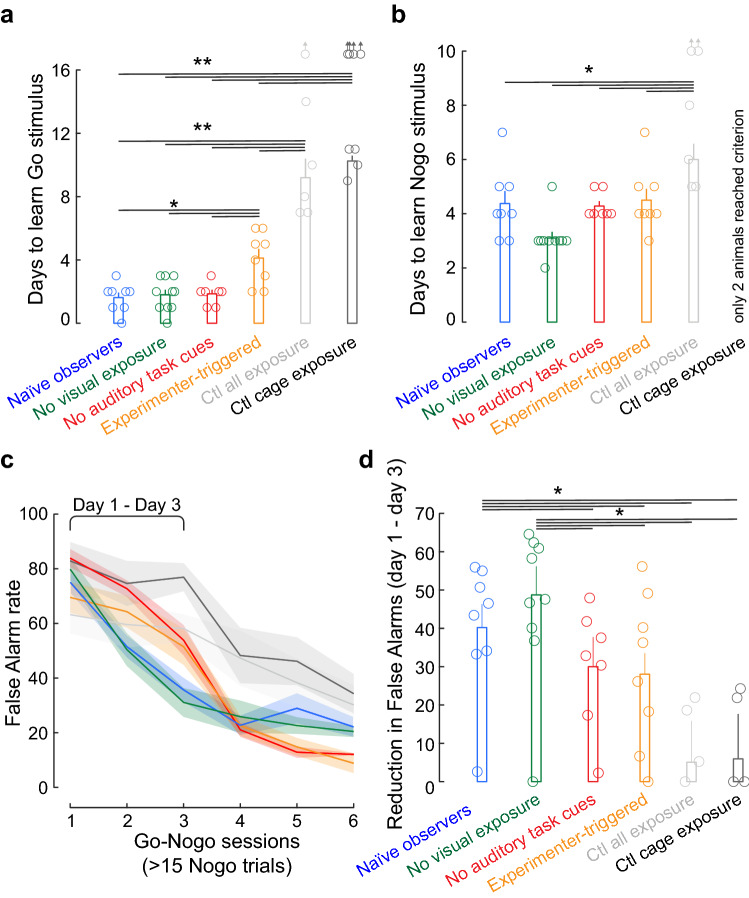
False Alarm rates decay faster for animals exposed to both social and auditory task cues. (**a**) Individual data (circles) and barplot showing the mean ± SEM number of practice days taken to perform > 25 Hits. The control group for social exposure was excluded as no animals performed > 25 Hits. The up-pointing arrows indicate that these animals did not perform > 25 Hits during the days tested. (**b**) Individual data (circles) and barplot showing the mean ± SEM number of days once all animals are performing > 15 Nogo trials to reach a criterion d’ of 1.5. (**c**) Mean ± SEM of the False Alarm rate decay as a function of practice sessions with > 15 Nogo trials. (**d**) Individual data (circles) and barplot showing the mean ± SEM improvement in False alarm rate from session 1 to session 3. Asterisks denote statistically significant post-hoc differences at the following levels: *p < 0.05 and **p < 0.01.

The control gerbils (Fig. 2a) who had prior social exposure did not learn the task, suggesting that social exposure alone was not sufficient to facilitate learning. To determine whether social exposure was necessary, we tested naïve gerbils that were exposed to a demonstrator performing a visual discrimination task, albeit with an opaque divider (Fig. 4a, top left). Thus, there were no auditory task cues present during the exposure sessions (i.e., Go and Nogo sound stimuli). However, the demonstrator’s motor behaviors were identical to those performed in the first two experiments (Figs. 1 and 3) . Following five daily exposure sessions, the gerbils experiencing no auditory task cues were permitted to practice the *auditory* discrimination task (Fig. 4a, top right). These gerbils (n = 7) required 6.1 ± 0.91 days to learn the task at a criterion d’ of 1.5, and their performance did not differ significantly from the naïve observer gerbils and the No visual exposure group (Kruskal–Wallis H test, X^2^(2) = 5.89, *p* = 0.053; BF_10_ = 0.5 and 2.9, respectively; see also Fig. 5a,b). This suggests that the facilitated task acquisition of the naïve observer gerbils and the No visual exposure group occurred largely through sensory cues of social origin.

We next asked whether the auditory task cues (i.e., Go and Nogo sound stimuli) were sufficient to facilitate task acquisition through statistical learning (see review^43^). To do so, a group of gerbils were exposed to only experimenter-triggered auditory task cues (Fig. 4b, top left). These included the task sound stimuli (i.e., Go and Nogo sound stimuli), and the response contingencies (i.e., pellet delivery to simulate Hits following a Go stimulus, time-outs to simulate False Alarms following a Nogo stimulus, as well as Misses and Correct Rejects). Following five exposure sessions, gerbils that were exposed to experimenter-triggered auditory task cues (n = 8) were permitted to practice the task (Fig. 4b, top right). The latter required 8.6 ± 1.85 days to learn the task at a criterion d’ of 1.5, and their performance was significantly worse than the naïve observer gerbils, the No visual exposure and the No auditory task cues groups (Kruskal–Wallis H test, X^2^(3) = 18.53, *p* = 0.0003, post-hoc comparisons, *p* < 0.05; BF_10_ = 12.8, 360.6, and 7.1, respectively; see also Fig. 5a,b). However, their performance was significantly better than the control groups shown in Fig. 2 (Kruskal–Wallis H test, X^2^(2) = 11.55, *p* = 0.003, post-hoc comparisons, *p* < 0.05, BF_10_ = 16.3 when comparing with control for cage exposure, and BF_10_ = 670.3, when comparing with control for all exposure; see also Fig. 5a,b). This suggests that exposure to the auditory task cues can facilitate task acquisition, even without social cues.

### Animals learn to discriminate more rapidly when exposed to both social and auditory cues

Following the exposure sessions, animals had to acquire two discrete behaviors: initiating a trial and obtaining a food reward within a 5 s window following a Go stimulus, and avoiding the food tray during a 5 s window following a Nogo stimulus. Here, we compared the rate of learning for each behavioral component. First, we compared the number of days taken to initiate and respond correctly to > 25 Go trials (Fig. 5a). The control groups (Fig. 2b,c) and the group exposed to experimenter-triggered auditory task cues (Fig. 4b) took significantly more days to complete this first learning phase, in comparison to all other groups (significant group effect: Kruskal–Wallis H test, X^2^(5) = 28.34, *p* < 0.0001, and significant post-hoc results, *p* < 0.05 are indicated with an asterisk in Fig. 5a). Next, we compared the number of days taken to reach a criterion d’ of 1.5 once animals were initiating > 15 Nogo trials (Fig. 5b). The control group for all exposure took significantly more days to reach the criterion d’ in comparison to other groups (Kruskal–Wallis H test, X^2^(4) = 19.63, *p* = 0.002, post-hoc comparisons, *p* < 0.05). The latter statistical comparison did not include the control group for cage exposure (Fig. 2b) because too few animals reached the criterion d’ of 1.5.

Next, we examined whether there were differences in learning strategies. Specifically, we asked whether there were group differences in how quickly the False Alarm rate declined across sessions with > 15 Nogo trials (Fig. 5c). Once animals begin to practice, their False Alarm rates are expected to decrease due to feedback (i.e., time-out period), but social learning could have hastened this process. Therefore, we calculated the difference between False Alarm rates during the initial practice sessions: session 1 versus session 3 (Fig. 5d). The naïve observer gerbils and No visual exposure group showed a significantly larger reduction in False Alarm rates, as compared to all other groups (Kruskal–Wallis H test, X^2^(5) = 16.35, *p* = 0.006, post-hoc comparisons, *p* < 0.05; BF_10_ = 4.5 and 8.7, respectively when comparing to the control group for all exposure). Thus, a faster reduction in False Alarm rate occurred only for the groups exposed to both the social cues (i.e., a demonstrator performing the task) and the auditory task cues (i.e., the Go and Nogo sounds). To confirm the robustness of this finding, a repeated measures ANOVA was performed (between subject factor: Group, repeated measures: False Alarm rate day 1, and False Alarm rate day 3). We observed a significant difference in False Alarm rates from day 1 to day 3 (F(1,35) = 64.776, *p* < 0.001), a significant Group effect (F(5,35) = 5.912, *p* < 0.001) and a significant interaction of Group and the repeated False Alarm rate measurement (F(5,35) = 4.683, *p* = 0.002).

### Interleaved exposure and practice does not necessarily improve the rate of learning

The practicing of motor skills immediately following exposure can be more effective than delayed practice^44,45^. Therefore, we tested the efficacy of interleaved practice in our paradigm by allowing gerbils to practice after each exposure session. In the first group, naïve gerbils were separated from a demonstrator by a transparent divider, and had access to all sensory cues (Supplementary Fig. 4a, top left). After each exposure session, the naïve gerbil was permitted to practice the task on its own (Supplementary Fig. 4a, top right). After the five days of interleaved exposure followed by practice, no further exposure session was carried out. The interleaved gerbil only practiced as from day 6. The rates of task acquisition did not differ significantly for naïve gerbils tested in an interleaved manner as compared to those tested only after five days of exposure (Steel–Dwass non-parametric comparisons, *p* = 0.417, see also Supplementary Fig. 4d). The interleaved naïve observer gerbils required 5.5 ± 0.43 days to learn the task at a criterion d’ of 1.5, while the delayed naïve observer gerbils (from Fig. 1) took 6.0 ± 0.93 days.

In the second interleaved group, naïve gerbils were separated from a demonstrator by an opaque divider, and did not have access to visual cues (Supplementary Fig. 4b, top left). After each exposure session, the naïve gerbil was permitted to practice the task on its own (Supplementary Fig. 4b, top right). After the five days of interleaved exposure followed by practice, no further exposure session was carried out. The interleaved No visual exposure gerbil only practiced as from day 6. In this cases, the rate of task acquisition was significantly better for the No visual exposure group tested in a delayed manner as compared to those tested in an interleaved manner (Steel–Dwass non-parametric comparisons, *p* = 0.008; see also Supplementary Fig. 4d). The interleaved No visual exposure group required 6.7 ± 0.29 days to learn the task at a criterion d’ of 1.5, while the delayed No visual exposure group (from Fig. 3) took 4.9 ± 0.4 days.

### Differences in learning rates were not explained by non-task factors

There were several experimental parameters that could have contributed to the findings, including sex and postnatal age of exposure and practice. No significant difference was found between male and female gerbils in terms of number of days to reach a criterion d’ of 1.5, when combined across groups (Kruskal–Wallis H test, X^2^(7) = 3.37, *p* = 0.910) or within each group (*p* > 0.05). Similarly, there was no sex difference in terms of best d’ performance attained when combined across groups (X^2^(1) = 3.47, *p* = 0.062), or within each group (*p* > 0.05). The mean postnatal age of the different groups differed significantly (Kruskal–Wallis H test, X^2^(7) = 36.82, *p* < 0.0001), with the group exposed to experimenter-triggered auditory task cues being significantly older than all other groups (Supplementary Fig. 5b, all post-hoc comparisons, *p* < 0.05). However despite being significantly older, the experimenter-triggered auditory task cues group performed a similar total number of trials, as compared to all other groups (with the exclusion of the control for social exposure group which did not learn the task; Kruskal–Wallis H test, X^2^(7) = 6.14, *p* = 0.408). The different groups did not differ significantly in terms of latency of response following trial initiation (Supplementary Fig. 5c; with the exclusion of the control for social and cage exposure groups which performed too few trials to be included, Kruskal–Wallis H test, X^2^(6) = 6.00, *p* = 0.423). No significant group difference was found in terms of total number of trials performed by the demonstrators during the 5 exposure sessions (Kruskal–Wallis H test, X^2^(5) = 9.05, *p* = 0.107). No significant correlation was found between the total number of trials the experimental groups were exposed to during the 5 exposure sessions and the total number of trials performed from practice day 1 to day 5 for all groups (Supplementary Fig. 5d; Spearman correlation, r = 0.11, *p* = 0.440). In addition, no significant correlations were found between the demonstrator’s performance (mean d’ across five days) and the exposed gerbil’s performance on any day of practice (Supplementary Fig. 5e shows the d’ on practice day 3; r = 0.14, *p* = 0.354). Together, these results suggests that there were no significant relationship between the number of trials performed by the demonstrator, nor the demonstrator’s performance and the rate of social learning. In addition, the differences in social learning rates shown in Fig. 5 could not be explained by any non-task factors.

## Discussion

Social learning occurs in a wide range of species and across many different natural environments. Here, we asked whether socially-mediated auditory and visual sensory cues were equally effective at facilitating the acquisition of an auditory discrimination task. We first showed that social learning was equally effective when all sensory cues were available (Fig. 1) and when visual cues were occluded (Fig. 3). Thus, naïve gerbils deprived of visual information were able to use other sensory cues (e.g., auditory) to facilitate social learning. Similarly, when visual cues and auditory task cues were eliminated, gerbils were still able to make use of socially-mediated sensory cues (Fig. 4a). Taken together, the experiments suggest that socially-mediated sensory cues are redundant and can be exploited by gerbils during social learning.

Task acquisition was significantly poorer when auditory task cues were experimenter-triggered, and all social cues were absent (Fig. 4b). This finding suggests that social cues are pivotal, and add to the physical, task-related cues, consistent with songbird studies in which the tutor is replaced by a recorded song^34,43,46,47^. The impact of socially-mediated cues was also illustrated by the control groups. The absence of any exposure prior to practicing the task was associated with significant delayed acquisition, relative to the social learning conditions (Fig. 2c). In fact, when naïve animals were exposed to non-performing cage mates, none of them were able to acquire the task over the course of 15 practice days (Fig. 2a). Thus, it is plausible that some forms of social experience can diminish the subsequent performance of a behavior.

In the present study, learning to perform the auditory discrimination task involved discrete behaviors, including trial initiation, seeking a reward following a Go stimulus, and re-poking following a Nogo stimulus. The acquisition of these elemental behaviors displayed a differential effect of social learning. For example, gerbils exposed to experimenter-triggered auditory task cues required a significantly greater number of practice session to initiate trials (Fig. 5a) but not in terms of learning the sound stimulus contingencies (Fig. 5b). In contrast, the control for all exposure group required a significantly greater number of days both to initiate trials and to learn the sound stimulus contingencies. For the naïve observer gerbil and No visual exposure groups, the False Alarm rate declined more rapidly in comparison to animals that were not exposed to the auditory task cues or that were exposed to the auditory task cues in a non-social manner (Fig. 5c,d). Therefore, naïve animals benefited most from the exposure sessions, when they experienced both socially-mediated and task-related sensory cues.

Our findings are also consistent with the environmental demands of rodents with underground burrows or nocturnal foraging behaviors, sea mammals living at depths with little light, and humans who lack visual experience. Like other rodents, gerbils have an elaborate repertoire of social behaviors^41^, including mutual grooming, vocal communication in both audible and ultrasonic ranges, dominance and appeasement poses, sexual behavior and pup-rearing. Since gerbils live in long underground burrows, and are particularly active at dawn or dusk^26–28^, the ability to use redundant sensory cues for social learning may be present in such species for which critical behaviors takes place under poor visibility. Indeed, gustatory-based social learning, common in gerbils^48^ and other species (humans^49^; monkeys^50^) can operate in the absence of visual exposure. Moreover, the finding that social learning is equally efficient in the auditory and visual domains is consistent with previous studies of vocal learning in songbirds^34,36,51^.

The social cues present in our paradigm could have taken several forms. At the sensory level, these include visual observation of the demonstrator’s actions, olfactory recognition of a conspecific, sharing of gustatory cues when naïve gerbils were returned to the home cage shared with the demonstrator or with other conspecifics that are also undergoing training, and acoustic spatial cues generated by a demonstrator’s movements. In fact, gerbils can form abstract representations of the environment when visual information is occluded^52^. Socially-mediated sensory cues could also have increased the naïve gerbil’s level of arousal, or increased the attentional resources during the exposure sessions^53^. Furthermore, rodents that are performing an operant task can learn to make choices that benefit a conspecific in a separate compartment, suggesting that social cues can have a profound effect on an animal’s motivation^54^. Future studies should attempt to tease apart the contributions of different types of social cues.

Social learning is present at most ages, but is particularly prevalent during development when juvenile animals spend extended time interacting with their siblings and parents. For example, juvenile rats spend more time engaged in social investigation, including nose- and body-sniffing, as compared to adults^55^. Young monkeys adopt the food preference of the mother^50^ and juvenile rats only ate foods they had observed elders eating previously and sampled food from the mouths of elders to acquire food preferences whereas elders sampled food from juveniles significantly less frequently^56^. Such forms of social learning is ubiquitous in human societies and widespread in many other species in the wild. For instance, young meerkats observe adults to learn how to handle prey^25^. Similarly, young chimpanzees learn nut-cracking techniques from experienced adults in their communities^18,57^ and young capuchin monkeys observe and learn from mature foragers that consume food requiring several processing steps^58^. Play behavior is also common in juvenile mammals (meerkats^59^; gerbils^60^; dolphins^61^), and can even be observed in reptiles^62^ and birds^63,64^. Play behavior occurs during the main period of physical, hormonal and social development of an animal and may thus provide short-term benefits to development^57,65,66^. Similarly, play behavior declines as animals mature, possibly because the benefits are lost^67,68^. In rodents, play-fighting behaviors peak before puberty and wane after puberty (rats^69,70^; hamsters^71^; gerbils^72^). In this study, we tested social learning in animals with a wide age range, from late adolescence to early adulthood. Future studies may assess whether juvenile animals display heightened social learning in comparison to the late adolescent and adult animals tested here.

The social learning literature has mainly focused on fear learning in rodents^17,73,74^ or spatial task learning in many species^5,13,29,30,75–77^, in which visual cues are presumed to play a central role. However, auditory cues permit animals to accurately localize a conspecific or another species^78–87^, even when the target is not visible. Here, we found that, in the absence of one modality of information, gerbils can still capitalize on the remaining information to learn in a social context. One plausible hypothesis that explains our results is that naïve gerbils can learn about the spatial behavior of demonstrators, either through visual or auditory signals, and can subsequently deploy this information when exploring and training on the auditory discrimination task. Thus, social learning is flexible and may, in part, explain its ubiquitous presence and function in many species across an array of environments.

## Methods

### Experimental animals

Gerbil (*Meriones unguiculatus*, n = 118) pups were weaned at postnatal day (P) 30 from commercial breeding pairs (Charles River). Littermates were caged together, but separated by sex, and maintained in a 12 h light/dark cycle. Same-sex litters were split into 2 subsets: 1. experimenter-trained demonstrator gerbils, and 2. naïve gerbils for exposure and practice. Each naïve animal was used for one single experimental or control paradigm. Data for each experimental or control paradigm was collected from at least 3 different litters in order to avoid any biases. Finally, animals in a given litter were used for at least 2 paradigms. All procedures related to the maintenance and use of animals were approved by the University Animal Welfare Committee at New York University, and all experiments were performed in accordance with the relevant guidelines and regulations.

### Behavioral setup

Gerbils were placed in a plastic test cage (dimensions: 0.25 × 0.25 × 0.4 m for 62 animals and 0.4 × 0.4 × 0.4 m for 42 animals) that was housed in a sound attenuation booth (Industrial Acoustics; internal dimensions: 2.2 × 2 × 2 m), and observed via a closed-circuit monitor. Auditory stimuli were delivered from a calibrated free-field tweeter (DX25TG0504; Vifa) positioned 1 m above the test cage. Sound calibration measurements were made with a ¼ inch free-field condenser recording microphone (Bruel & Kjaer). Visual stimuli were delivered with a LED (Sparkfun) positioned on top of the cage. A pellet dispenser (Med Associates Inc, 20 mg) was connected to a food tray placed within the test cage, and a nose port was placed on the opposite side. The nose port and food tray were equipped with IR emitters and sensors (Digi-Key Electronics; Emitter: 940 nm, 1.2 V, 50 mA; Sensor: Photodiode 935 nm 5 nS). Stimuli, food reward delivery, and behavioral data acquisition were controlled by a personal computer through custom MATLAB scripts and an RZ6 multifunction processor (Tucker-Davis Technologies).

Videos of the test cage were captured with a Logitech c270-HD webcam (30 frames per second, Best Buy). We used the open source software: DeepLabCut^88^ on a Windows 10 machine (Dell Precision 5,820, 64-bit operating system) to track the gerbil’s position in the test cage during practice sessions. The network was trained with 1,030,000 iterations using a total of 1,064 labeled frames (labeling of nose, left ear, right ear, and tail base) and tested on a set of 200 frames. Manual evaluation of labeling accuracy was achieved by comparing the labels acquired from the network on the test set with the manual labels.

### Stimuli

For the sound discrimination task, the Go stimulus consisted of amplitude modulated (AM) frozen broadband noise tokens (25 dB roll-off at 3.5 kHz and 20 kHz) with a modulation rate of 12 Hz and a modulation depth of 100%. The sound level used was 66 dB SPL. The Nogo stimulus was similar to the Go stimulus, except for the modulation rate which was 4 Hz. Both Go and Nogo stimuli had a 200 ms onset ramp, followed by an unmodulated period of 200 ms which then transitioned to an AM stimuli.

For the visual discrimination task, the Go stimulus consisted of a blue LED (wavelength: 400–465 nm, brightness: 2,500–3,000 MCD) which was modulated at 12 Hz and a modulation depth of 100%. The Nogo stimulus was similar to the Go stimulus but was modulated at 4 Hz. Similar to the sound stimuli, a 200 ms onset ramp was used, followed by an unmodulated period of 200 ms before transitioning to modulated light.

### Experimenter trained demonstrator gerbils

Demonstrator gerbils were trained by the experimenters on either a sound discrimination task (n = 37, age = 76.1 ± 21.15), or a visual discrimination task (n = 6, age = 63.0 ± 2.45). Both groups of demonstrators were placed on controlled food access two days prior to the start of training, and all animals were trained using an appetitive reinforcement operant conditioning procedure (see Supplementary Fig. 1). When introduced to the test cage, animals first learned to eat food pellets (Bio Serv) placed in the food tray (stage 1). After this phase, the Go stimulus (12 Hz AM) was delivered whenever animals were at the food tray (stage 2). Animals were then trained to respond to the Go stimulus by approaching the food tray (stage 3). After this sound-food (or light-food) association phase, the nose port was placed in the testing cage. During the first day of nose port training, the experimenter triggered trials through the computer whenever animals were in close proximity to the port (stage 4). This maximized exploration of the nose port and facilitated poking behavior. Within one to three training sessions, animals were shaped to reliably initiate Go trials independently by placing their nose in the port. During the nose port training, only Go trials were presented. Once animals reached a hit rate > 80% and were performing a minimum of 80 Go trials, Nogo trials were introduced (stage 5). The probability of Nogo trials was kept at 30% in order to keep the animal motivated to perform the task.

During Go trials, responses were scored as a ‘Hit’ when animals approached the food tray to obtain a food reward. If animals re-poked or did not respond during the 5-s time window following a Go stimulus, then it was scored a ‘Miss’. During Nogo trials, responses were scored as a ‘False Alarm’ when animals incorrectly approached the food tray. If animals re-poked or did not respond during the 5-s time window following a Nogo stimulus, then it was scored a ‘Correct Reject’. On the second day of Nogo training, False Alarm trials were paired with a 2-s time-out during which the house lights were extinguished and the animal could not initiate a new trial. From day 3 onwards, a 4-s time-out was used when animals False Alarmed. The presentation of Go and Nogo trials were randomized to avoid animals developing a predictive strategy. A performance metric^89^, d prime (d’) was calculated for each session by performing a z-transform of both Hit rate and False Alarm values: d' = z(Hit rate) – z(False Alarm rate). To qualify as a *demonstrator*, animals were required to perform the task with a d’ > 1.5 for 3 consecutive days. This experimenter-training procedure took on average 15.9 days ± 0.17 days (see Supplementary Fig. 1b,c).

### Social learning paradigms

During each *exposure session*, a demonstrator gerbil performed the discrimination task in the presence of a naïve, untrained gerbil that was a same-sex cage mate. All pairs of demonstrator and observer animals were thus housed together prior to any training and during the exposure and practice sessions. Both the demonstrator and the naïve gerbil were placed on controlled food access. A divider (acrylic sheet) was placed within the test cage to separate the demonstrator compartment from the observation or exposure compartment (see Fig. 1a). A nose port and food tray were present only on the demonstrator’s compartment, allowing the demonstrator to initiate and perform trials. The naïve gerbil was exposed to a minimum of 60 Go trials and 20 Nogo trials in each exposure session. All demonstrators were performing trials with a mean Hit rate of 96.0% ± 1.46 and a mean False Alarm rate of 27.15% ± 6.78 across all the five days of exposure.

For the initial experiments, there were five daily exposure sessions. The divider was removed after the final day of exposure, and the naïve gerbil was then allowed to practice the task (*practice session*). During the first and second practice sessions, the latter was given the benefit of no more than five experimenter-triggered Go trials. These experimenter-triggered Go trials were initiated only when an animal was touching the nose port. This method of manually initiating Go trials was identical to the one used to train demonstrators, in order to maximize the animal’s interest in the nose port object. Except for these experimenter-triggered Go trials, all Go trials were initiated by the gerbil. Once an animal was reliably initiating Go trials and performed > 25 Hits, we introduced Nogo trials. False Alarm trials were paired with a 2-s time-out on the second day of Nogo trial introduction. For all following practice days, a 4-s time-out was used when animals False Alarmed. A d’ was computed for all practice sessions during which a minimum number of 15 Nogo trials were presented (Fig. 1b,c).

Naïve gerbils (n = 75, age = 96.81 ± 32.69) were tested on one of the following paradigms. No animals were excluded from the study.

*Naïve observer* gerbils were separated from a demonstrator by a transparent divider, allowing access to all sensory cues (Fig. 1a, left). Although no attempt was made to coerce observation, the naïve gerbil was in a position to watch the performing demonstrator as well as the experimental apparatus (food tray and nose port). Following five, daily exposure sessions, the *naïve observer gerbil* was permitted to practice the task (Fig. 1a, right).

*No visual exposure* gerbils were separated from a demonstrator by an opaque divider, blocking access to visual cues (Fig. 3a, left). However, these No visual exposure animals maintained access to auditory, tactile, and olfactory cues. Following five, daily exposure sessions, the *No visual exposure* animal was permitted to practice the task (Fig. 3a, right).

*No auditory task cues* animals were separated from a demonstrator that was performing a visual discrimination task (Fig. 4a, left). Visual access to the demonstrator and the LED stimuli were restricted with an opaque divider. In addition, the naïve gerbils were not exposed to the auditory task cues (i.e., the Go and Nogo sound stimuli). Following five, daily exposure sessions, the *No auditory task cues* gerbil was permitted to perform the auditory discrimination task (Fig. 4a, right).

*Experimenter-triggered auditory task cues* animals were separated from an unoccupied demonstrator compartment by an opaque divider (Fig. 4b, left). The experimenter triggered Go and Nogo stimuli from outside the test cage, as well as time-outs during which house light were extinguished to simulate False Alarms, and delivery of pellets into the food tray to simulate Hits. Misses following Go trials and Correct Rejects following Nogo trials were also triggered. Thus, during the exposure sessions, the *experimenter-triggered auditory task cues* gerbil retained access to the Go and Nogo sound stimuli, and all task contingencies in a non-social manner. Following five, daily exposure sessions, the *experimenter-triggered auditory task cues* gerbil was permitted to perform the task (Fig. 4b, right).

*Control for social exposure* animals were separated from a naïve, untrained and non-performing cage mate by a transparent divider, and the nose port and food tray were removed from its compartment to avoid accidental trial initiation (Fig. 2a, left). Following five daily social exposure sessions, the *Control for social exposure* animal was permitted to perform the task for 15 daily sessions (Fig. 2a, right).

Control for cage exposure animals were separated from an unoccupied demonstrator compartment by a transparent divider (Fig. 2b, left), and were exposed to five daily sessions in the test cage prior to practice. The nose port and food tray were kept in place in the demonstrator’s compartment. Following five daily exposure sessions, the *Control for cage exposure* animal was permitted to perform the task for 15 daily sessions (Fig. 2b, right).

*Control for all exposure* animals were permitted to perform the auditory discrimination task for 15 daily sessions (Fig. 2c). These animals received no prior social exposure with a cage mate in the test cage, nor any exposure to the test cage itself.

An additional control was performed to test whether familiarity with the demonstrator through sharing of a home-cage facilitated social learning. Naïve observer gerbils were separated from a demonstrator by a transparent divider for five daily exposure sessions, after which the animals were permitted to perform the task, similarly to Experiment 1. However in this case, the naïve observer gerbils were paired with non-familiar, same-sex demonstrators during the exposure sessions (see Supplementary Fig. 3). Thus, in this particular control condition, the demonstrators and the observers did not share a home cage.

Finally, two additional groups of animals were tested in an *interleaved paradigm*. The Interleaved animals were permitted to practice the auditory task following each exposure session. After five daily exposure sessions, each of which was followed by practice, animals received no further exposure, but continued daily practice. The first interleaved group was separated from the demonstrator by a transparent divider and the second interleaved group was separated from the demonstrator by an opaque divider (see Supplementary Fig. 4).

### Performance measures and statistical analyses

Due to limited litter sizes, a given litter could not be split into our nine experimental and control conditions. However, animals from one given litter was used for at least 2 conditions, with half of them being trained as demonstrators and the other half used as naïve exposed animal. In addition, for all groups, data was collected from at least 3 different litters in order to avoid any litter-specific biases. No litter differences were observed during the experimenter-training stages of the demonstrator animals. A performance measure^89^ (d’) was calculated for each animal: d' = z(Hit rate) – z(False Alarm rate). Hit and False Alarm rates were constrained to floor (0.05) and ceiling (0.95) values. A d’ was computed for every session during which the gerbil performed > 15 Nogo trials. For the computation of the mean d’ line, we used all values of d’ and attributed a zero to all NaN values of d’ (i.e., when an animal was initiating < 15 Nogo trials, hence no d’ value could be computed). However, for all statistical tests and for the computation of the mean number of days taken by each experimental and control group to reach a criterion d’ of 1.5, only actual d’ values were used. We first checked whether the number of days for each group to reach a criterion performance d’ of 1.5 were normally distributed using the Shapiro Wilk test of normality. As the latter were not sufficiently Gaussian, we chose to perform non-parametric tests. All group level statistical tests and effect size calculations were performed using JMP Pro 14.0 on a Mac platform. Pairwise comparisons were carried out using the Steel–Dwass Method for non-parametric comparisons. To compare more than 2 groups, one-way ANOVA rank tests (Kruskal–Wallis H test) were used. For post-hoc multiple comparisons analyses, alpha values were Holm-Bonferroni-corrected. In addition, confirmatory Bayesian statistical analyses were computed with the software JASP using default priors^90^. We reported Bayes factors (BF_10_), which reflect how likely the data is to arise from one model, compared, in our case, to the null model (i.e. the probability of the data given H1 relative to H0). Bayesian one-way ANOVAs, followed by post-hoc Bayesian independent samples t-tests corrected for multiple comparisons were computed.

## Supplementary information

Supplementary file1

Supplementary file2

## Data Availability

Behavioral source data are available upon request at https://nyu.app.box.com.
