# Supplementary material for: Social learning exploits the available auditory or visual cues
Source: Sci Rep. 2020 Aug 24;10:14117. doi: 10.1038/s41598-020-71005-x (PMC7445250; doi:10.1038/s41598-020-71005-x)
Supplement: Supplementary file 2 — Supplementary file2 [file 41598_2020_71005_MOESM2_ESM.pdf]

Title **Social learning exploits the available auditory or visual cues**

Authors **Nihaad Paraouty, Joey A. Charbonneau, Dan H. Sanes**

Supplementary Information

Supplementary Figures 1-5 and Figure legends

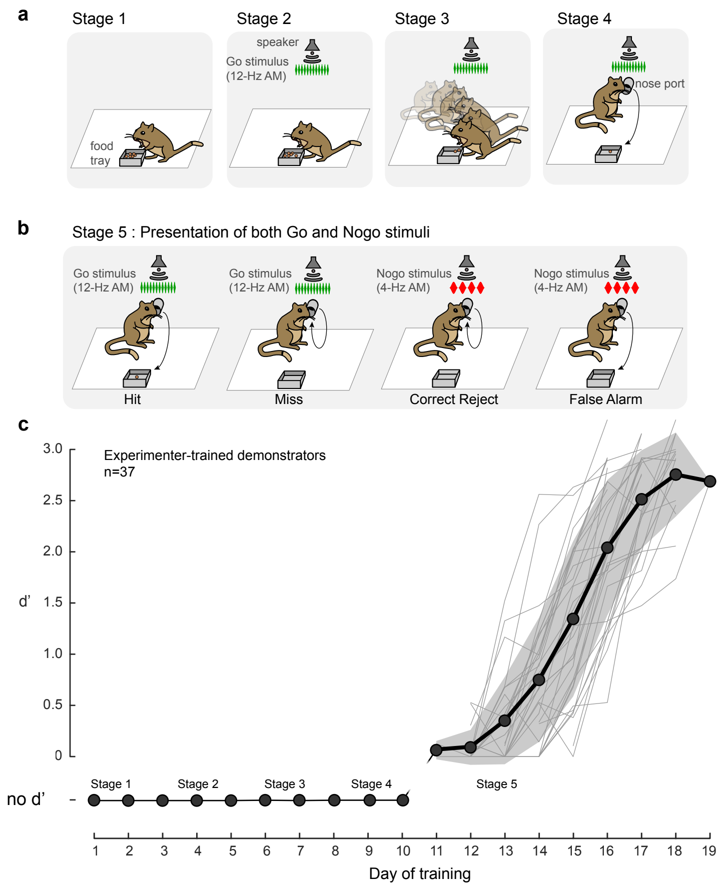

Supplementary Figure 1

Experimenter-training stages for the demonstrator gerbils. **a.** Stage 1: Test cage habituation. Naïve gerbils are placed on controlled food access, and they learn to eat food pellets from a food tray in the test cage. To proceed to the next stage, gerbils must be eating > 50 pellets. For the 37 demonstrators trained, this stage took on average 2.5 ± 1.8 days. Stage 2: sound (or light) habituation using the Go stimulus (12-Hz AM noise or 12-Hz LED pulse). Gerbils next learn to eat with the Go stimulus on. To proceed to the next stage, gerbils must be eating > 50 pellets. This stage took on average 2.2 ± 0.5 days. Stage 3: sound (or light) and food association. Animals learn to run to food tray upon Go stimulus onset. To proceed to the next stage, gerbils must be performing > 50 Hit trials. This stage took on average 3.2 ± 1.3 days. Stage 4: nose-poking training. Animals learn to initiate trials by entering their nose in the nose port and running to food tray upon Go stimulus onset. To proceed to the next stage, gerbils must be performing > 80 Hits. This stage took on average 3.4 ± 1.0 days. **b.** Stage 5: Go-Nogo discrimination task. Nogo stimuli are introduced and animals learn to discriminate the 12-Hz versus the 4-Hz AM noise (or LED pulse). If the animal goes to the food tray for the Go stimulus, it is considered a Hit. If the animal repokes on a Go stimulus or does not go to the food tray in the 5-second time window, it is considered a Miss. If the animal repokes on the Nogo stimulus, it is considered a Correct Reject. If the animal goes to the food tray on the Nogo stimulus, it is considered a False Alarm and a time-out is given. **c.** A performance measure, d' is computed with d’= z(Hit rate) – z(False Alarm rate). The d’ is shown across training days. .

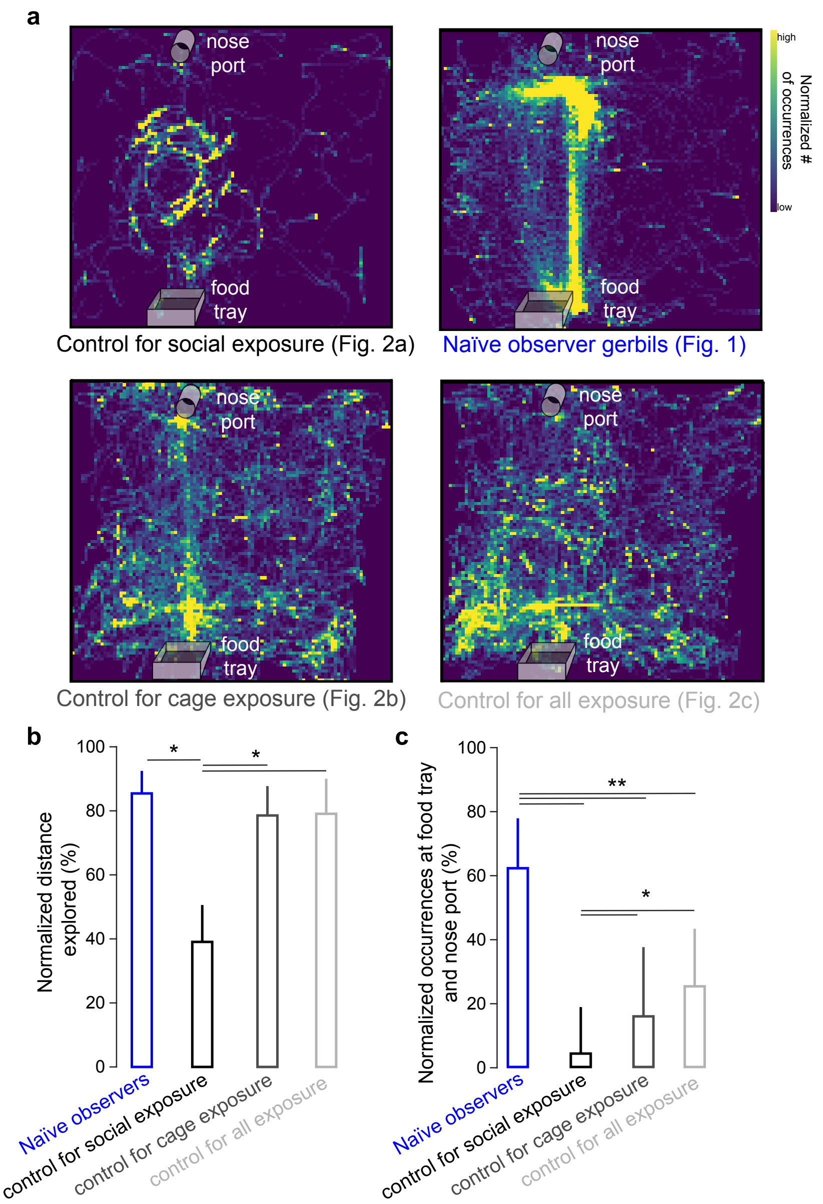

Supplementary Figure 2

Uneven exploration of test cage during practice sessions. **a.** The x and y coordinates corresponding to the nose position of a given animal during the practice sessions were extracted using the open source software, DeepLabCut (see Methods for details). Example heatmaps are shown on practice day 5 for a naïve observer gerbil (blue) and three control conditions. **b.** Mean ± SEM of the total Euclidean distance travelled during practice session 5 for a subset of animals from each group (n=3). The total distance was normalized for each given animal by the total number of frames in the given session. **c.** The exploration of the nose port and the food tray was assessed by taking the normalized number of occurrences of the x and y coordinates of the nose of the animal within ~2 cm of each object. Asterisks denote statistically significant post-hoc differences at the following levels: * $p<0.05$ and ** $p<0.01$.

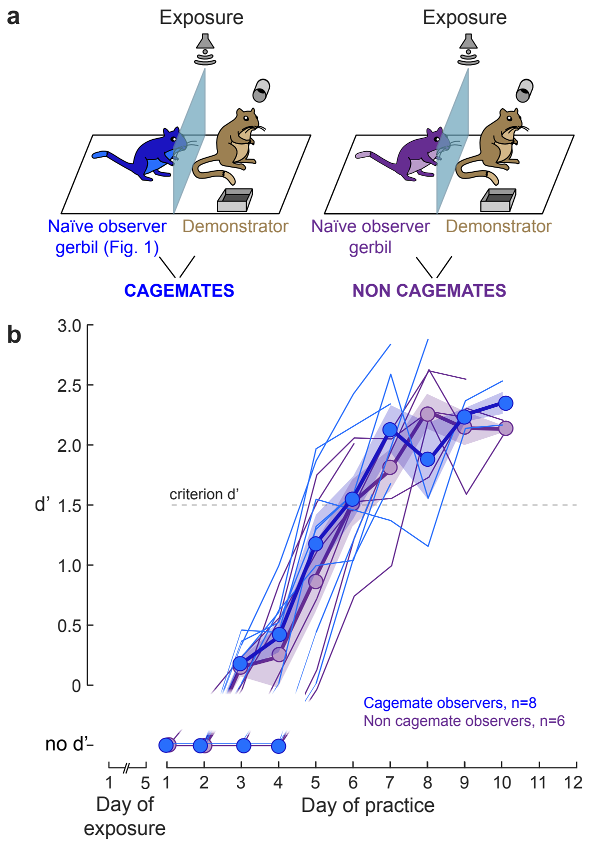

Supplementary Figure 3

Familiarity with a demonstrator does not affect social learning. **a.** Left: Experimental design 1 (from Fig. 1) in which naïve gerbils are exposed to a cagemate demonstrator for 5 daily exposure sessions. Thus, both naïve gerbils and demonstrator animals share a home cage prior to and during the duration of the experiment. Right: Experimental design 1, but naïve gerbils are exposed to a non-cagemate, and non-familiar demonstrator animal for the 5 daily exposure sessions. In this case, the naïve gerbils are not housed in the same home cage as their respective demonstrator animals. **b.** Individual (thin lines) and mean ± SEM (thick lines) performance d' values of the two groups of naïve observer gerbils during the practice sessions (blue for cagemates and purple for non-cagemates).

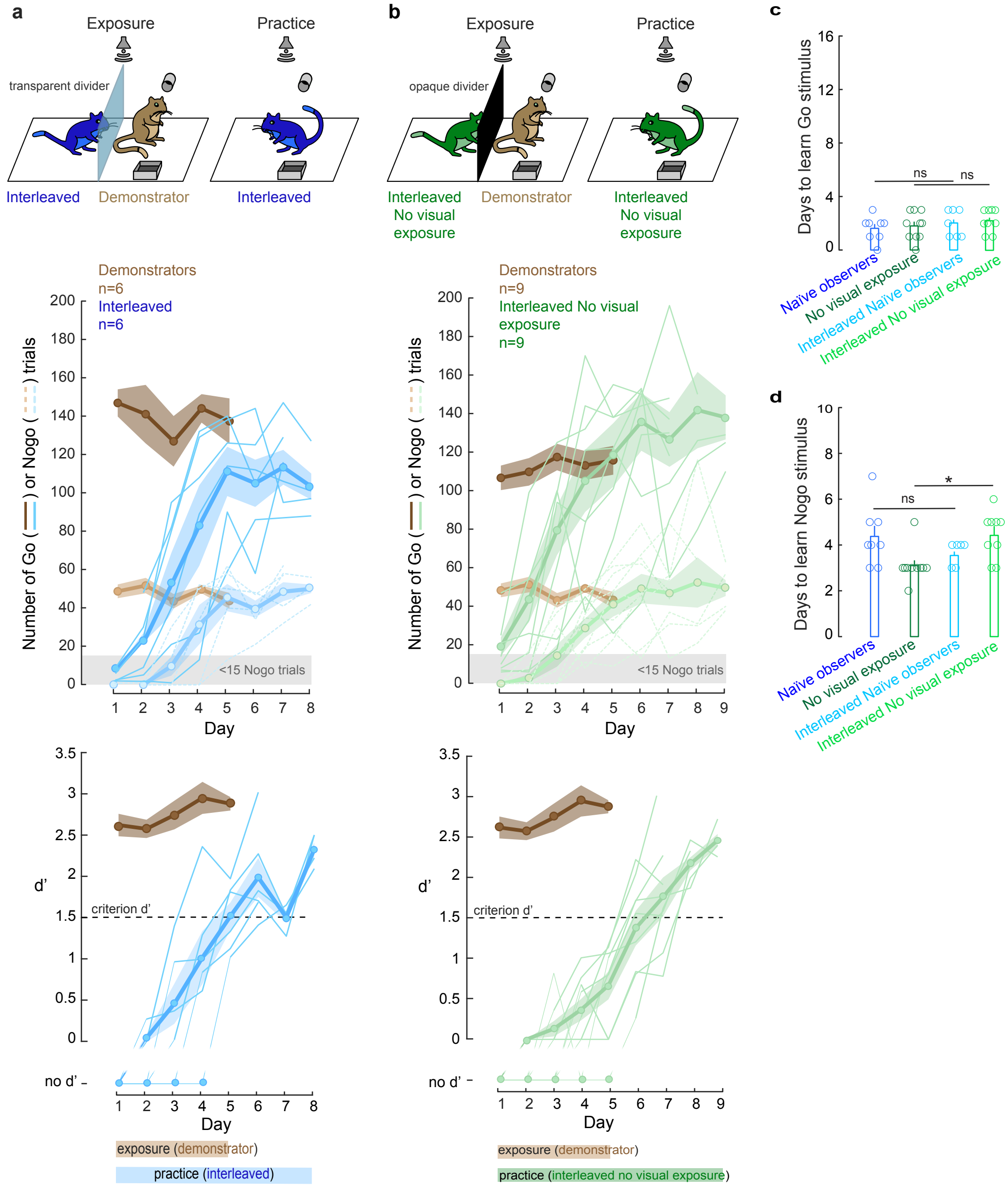

Supplementary Figure 4

Daily practice following exposure does not necessarily improve rate of task acquisition. **a.** Experimental design 1, with interleaved exposure and practice: A group of naïve gerbils was ran similar to the naïve observer gerbils from Fig. 1, except that after each exposure session, the interleaved animal was given a practice session. After the five days of interleaved exposure followed by practice, no further exposure session was carried out. The interleaved animals only practiced as from day 6. **b.** Experimental design 2, with interleaved exposure and practice: A group of naïve gerbils was ran similar to the No visual exposure from Fig. 3, except that after each exposure session, the No visual exposure animal was given a practice session. After the five days of interleaved exposure followed by practice, no further exposure session was carried out. The interleaved No visual exposure animal only practiced as from day 6. **c.** Individual data (circles) and barplot showing the mean ± SEM number of practice days taken to perform > 25 Hits for the interleaved and delayed practice animals. **d.** Individual data (circles) and barplot showing the mean ± SEM number of days once all animals are performing > 15 Nogo trials to reach a criterion d' of 1.5.

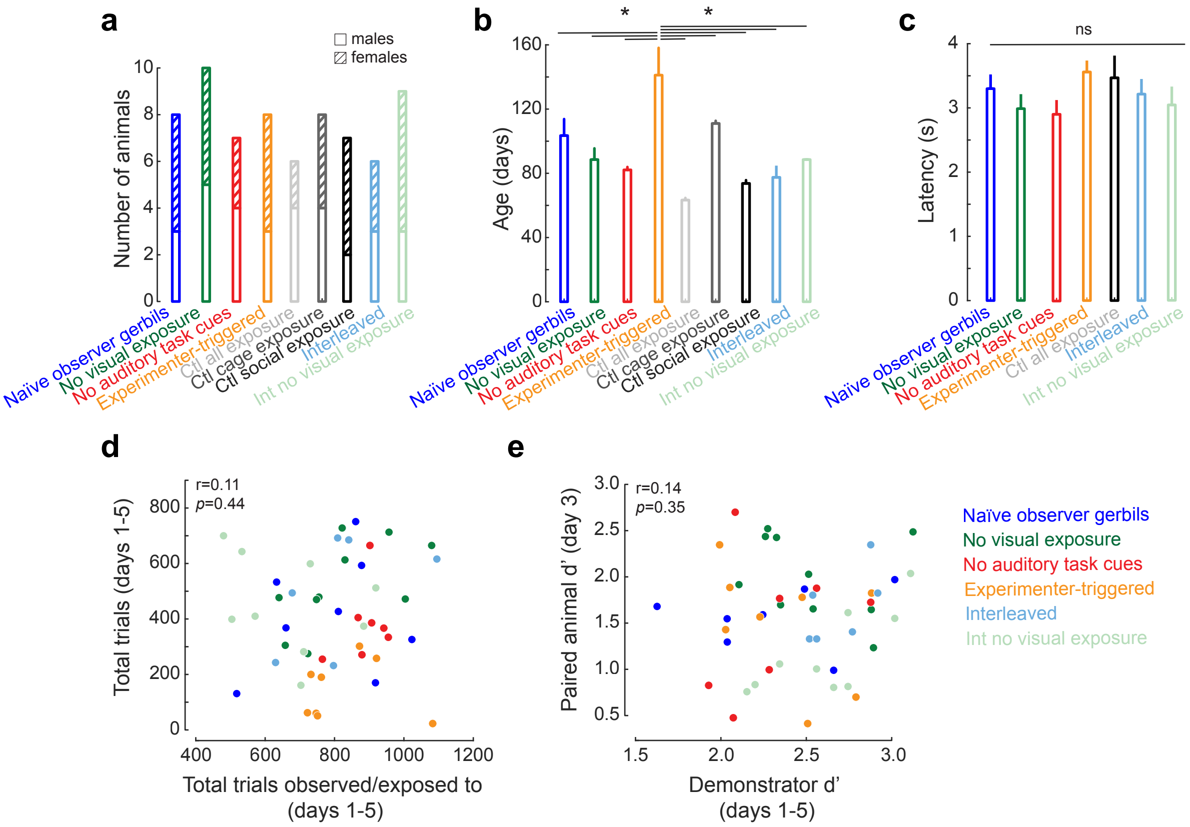

Supplementary Figure 5

Learning differences are not accounted by non-task factors. **a.** The number of male and female gerbils are shown for all groups tested. **b.** The postnatal age on the first day of practice for the different groups. **c.** The latency of response for the different groups for sessions with > 15 Nogo trials. **d.** The total number of trials performed by the demonstrators during the 5 exposure sessions are shown against the total number of trials practiced from day 1 to day 5 for all experimental groups. **e.** The demonstrators' mean d' across the 5 exposure sessions are shown against the exposed animals' d' on day 3 of practice.
